# Efficacy of oral, topical and extended-release injectable formulations of moxidectin combined with doxycycline in *Dirofilaria immitis* naturally infected dogs

**DOI:** 10.1186/s13071-023-05673-9

**Published:** 2023-02-06

**Authors:** Lavinia Ciuca, Alice Vismarra, Dario Constanza, Antonio Di Loria, Leonardo Meomartino, Paolo Ciaramella, Giuseppe Cringoli, Marco Genchi, Laura Rinaldi, Laura Kramer

**Affiliations:** 1 Department of Veterinary Medicine and Animal Production, University of Naples Federico II, Via Federico Delpino 1, 80137 Naples, Italy; 2 Department of Veterinary Medicine Sciences, University of Parma, Strada del Taglio, 10, 43126 Parma, Italy

**Keywords:** *Dirofilaria immitis*, Moxidectin, Doxycycline, Spot-on, Oral, Injectable

## Abstract

**Background:**

Several studies in both experimentally and naturally infected dogs have reported the adulticide effect of a combination of macrocyclic lactones and doxycycline against *Dirofilaria immitis*, showing that these protocols can be used as an alternative to melarsomine. The present study evaluated the efficacy of oral, topical and extended-release injectable formulations of moxidectin when combined with doxycycline in dogs naturally infected with *D. immitis* from a shelter located in southern Italy.

**Methods:**

Thirty dogs with naturally acquired *D. immitis* infection were divided in three groups (G) and treated with oral moxidectin (G1) once a month for 9 consecutive months, topical moxidectin (G2) once a month for 9 consecutive months or extended release moxidectin injectable (G3) at enrolment and again at 6 months (Day 180). All treatment groups received doxycycline for the first 30 days. Microfilarial concentrations in 1 ml (mff/ml) blood were determined monthly for 9 months with the modified Knott’s test. A clinical scoring system was employed for each dog enrolled in the study based on thoracic radiography and cardiac ultrasound (CU) examinations performed at Day − 15 (before treatment) and at Day 180.

**Results:**

Results from the present study suggest that the majority of dogs from all treatment groups became antigen negative, as evaluated at Day 270: 9/10 dogs (90.0%) from G1, 6/10 dogs (60.0%) from G2 and 8/10 dogs (80.0%) from G3. Improvement of radiographic alterations was observed in all treatment groups, and almost all dogs were cleared of pulmonary abnormalities by 6 months from the beginning of treatment (*P* = 0.000). Cardiac ultrasound examination showed a progressive improvement of cardiac function in a limited number of animals (4/30).

**Conclusions:**

The combination of doxycycline and three different formulations of moxidectin leads to antigen-negative status in naturally infected dogs.

**Supplementary Information:**

The online version contains supplementary material available at 10.1186/s13071-023-05673-9.

## Background

Canine heartworm disease (HWD) is caused by the filarial nematode *Dirofilaria immitis*, a vector-borne parasite transmitted by several mosquito species, and is endemic in many parts of the world [1]. The presence of adult worms in the pulmonary arteries of infected dogs causes changes in arterial structure and function that can lead to pulmonary hypertension and, eventually, to right-sided congestive heart failure [2, 3]. Melarsomine dihydrochloride is the only approved adulticidal drug for treatment of HWD. Several studies in both experimentally and naturally infected dogs have reported the adulticide effect of a combination of macrocyclic lactones (ML) and doxycycline against *D. immitis*, showing that these protocols are safe and effective [4–11]. Doxycycline targets the bacterial endosymbiont *Wolbachia*, whose reduction contributes to worm infertility and death [12, 13]. This activity, combined with the known detrimental effects of ML, may eliminate adult worms and lessens the inflammatory reaction against dead and dying worms [14]. The American Heartworm Society [15] and the European Society of Dirofilariosis and Angiostrongylosis [16] currently suggest that in cases where treatment with melarsomine is not possible or is contraindicated, a monthly treatment based on ML along with doxycycline for a 4-week period might be considered [11, 15, 16].

It has been shown that the combination of moxidectin/doxycycline has superior adulticide efficacy compared to ivermectin/doxycycline [17]. Grandi et al. [5] reported 73% efficacy 10 months after the beginning of doxycycline (daily for 1 month) combined with oral ivermectin every 15 days for 6 months. More recently, Savadelis et al. [8] reported a 95.9% adulticide efficacy in experimentally infected dogs after 1 month of doxycycline combined with 10 monthly treatments with a topical formulation of moxidectin. Genchi et al. [9] reported that 15/16 naturally infected dogs became antigen negative at 9 months with the same protocol. Paterson et al. [10] reported 93.0% of dogs treated with the same protocol were antigen-free at month 15. There is only one report on the efficacy of extended-release injectable moxidectin combined with doxycycline in naturally infected dogs, in which 90.0% of the dogs were antigen negative within 1 year of treatment with 1 month of doxycycline and administration of two doses of injectable moxidectin at 6 and 12 months [18]. To the authors' knowledge, there are no published data regarding the evaluation of oral moxidectin combined with doxycycline.

The aim of the present study is to evaluate the effect on antigen status of oral, topical and extended-release injectable formulations of moxidectin when combined with doxycycline in dogs naturally infected with *D. immitis* from a shelter located in southern Italy.

## Methods

### Animals

**Fig. 1:**
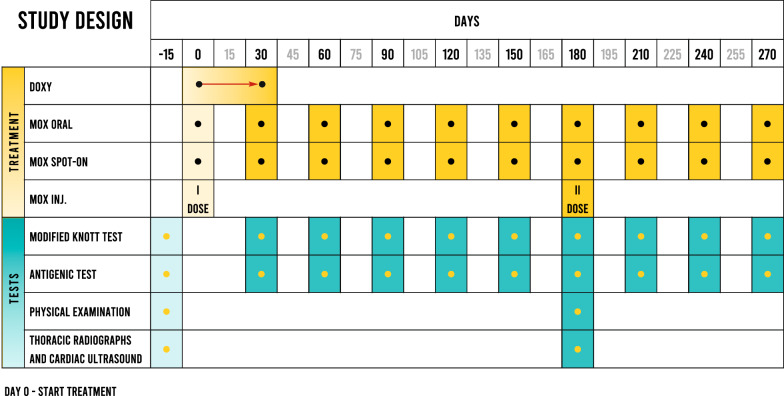
Study design. DOXY = 10 mg/kg SID (Ronaxan®, Boehringer Ingelheim Animal Health, Italy); MOX ORAL = oral moxidectin (3 µg/kg SID; Afilaria®, Fatro-Italy); MOX SPOT-ON = topical 10% imidacloprid + 2.5% moxidectin (Advocate®, Elanco, Italy); MOX INJ = extended release moxidectin injectable (Afiliara SR®, Fatro, Italy)

The study started in July 2020 and ended September 2021 (Fig. 1). The protocol of this study was approved by the ethics committee of animal experiments of the Department of Veterinary Medicine and Animal Production, University of Federico II Naples, Italy (approval number 0093204/2022). Shelter owner’s consent was obtained prior to the start of the study.

Thirty dogs with naturally acquired *D. immitis* infection were enrolled from a municipal shelter located in southern Italy (Castel Volturno, Campania region; geographical coordinates 41°0′57″24 N and 13°56′49″56 E). Inclusion criteria were dogs of any breed/sex, weighing at least 1 kg body weight, being > 6 months of age and not having been treated within 2–3 months prior with any macrocyclic lactone or doxycycline.

Dogs were considered positive for infection when a circulating antigen test (PetChek® HTWM PF, IDEXX, USA) and/or microfilariae (mff; modified Knott’s test) was positive for *D. immitis.* According to the manufacturer’s instructions, all the samples were tested in duplicate. A sample was considered positive if it had more colour than the negative control (see “Parasitological evaluation” section for the scoring system, based on the intensity of the colour reaction).

### Treatment protocols

The 30 dogs were allocated at random into three treatment groups (10 dogs in each group), based on the moxidectin formulations used for the treatment protocols as described below.

Group 1 (G1). Oral moxidectin (3 µg/kg SID; Afilaria®, Fatro-Italy) once a month for 9 consecutive months, together with doxycycline (10 mg/kg SID; Ronaxan®, Boehringer Ingelheim Animal Health, Italy) for the first 30 days.

Group 2 (G2). Topical 10% imidacloprid + 2.5% moxidectin (Advocate®, Elanco, Italy) once a month for 9 consecutive months, together with doxycycline (10 mg/kg SID; Ronaxan®) for the first 30 days.

Group 3 (G3). Extended release moxidectin injectable (Afiliara SR®, Fatro, Italy) every 6 months for two administrations, together with doxycycline (10 mg/kg SID; Ronaxan®, Boehringer Ingelheim Animal Health, Italy) for the first 30 days.

### Parasitological evaluation

Blood samples were collected once a month for 9 months from all dogs enrolled in the study. Microfilarial concentration in 1 ml (mff/ml) blood was determined with the modified Knott’s test [19] following the protocol by Genchi et al. [20]. Morphological identification of microfilariae was based on body length and width and the morphology of the head and tail [20]).

Serum samples were tested for circulating antigens using PetChek® HTWM PF (IDEXX, USA), following the manufacturer’s instructions. A subjective scoring system, based on the intensity of the colour reaction, was used to express the results of each test as negative (−), weak positive (+) and strong positive (+ +). “First negative” was defined as a minimum of two consecutive negative tests, according to [10].

### Thoracic radiography

The radiological evaluation was performed at Day − 15 (before the treatment) and at Day 180 (after 6 months of treatment) by the same operator.

The thoracic radiographs included right-lateral, left-lateral and dorso-ventral views. All the radiographs were obtained on manually restrained and unsedated patients during peak inspiration. The following criteria were evaluated: size and shape of the pulmonary arteries and their branches (vascular pattern); dilatation of the main pulmonary artery, enlargement of the right atrium, enlargement of the left atrium, generalized cardiac enlargement evaluated both subjectively and objectively using the vertebral heart score (VHS) [21]. Furthermore, the presence and distribution of alterations affecting the pulmonary interstitium, alveolar space and bronchial walls were classified using common radiographic patterns: interstitial, alveolar and bronchial pattern, respectively, or mixed if two or more patterns were present [22].

Based on the presence and degree of extension of one or more of the radiographic alterations, a clinical scoring system was considered as follows: score 0 (normal, without radiographic alterations consistent for heartworm disease), score 1 (mild pulmonary and/or cardiac alterations), score 2 (moderate pulmonary and/or cardiac alterations) and score 3 (severe pulmonary and/or cardiac alterations, signs compatible with thrombo-embolism/pneumonia) [6, 23].

### Cardiac ultrasound

All the dogs underwent complete echocardiographic examination at Days − 15 and 180 by the same operator, including transthoracic 2D, M-mode and spectral and colour-flow Doppler echocardiographic evaluations using transducer arrays of 1–4 MHz and 3–8 MHz (ESAOTE, Italy).

Examinations were performed in conscious unsedated dogs during a period of quiet breathing, with the animals in left and right lateral recumbency. At least five consecutive cardiac cycles were acquired and stored for off-line measurements. A clinical scoring system was employed based on ultrasound findings, according to [6, 24, 25]. Briefly, pulsed wave (PW)-spectra signals for calculation of STIs were acquired from the right parasternal short axis view of the pulmonary artery with the sample volume at the valve level; the acceleration time (AT) of pulmonary artery (PA) flow was measured from the onset of the pulsed Doppler PA flow signal to peak flow velocity. The right ventricular ejection time (ET) was measured from the onset to the end of the Doppler PA flow signal; moreover, the AT/ET ratio was calculated. When tricuspid regurgitation and/or mitral regurgitation were present, CW spectra were acquired from the left apical cranial four-chamber views. Each dog was assigned a score from 0 to 3 for pulmonary hypertension, considering AT, ET, AT/ET and TRV [6].

Radiological and echocardiographic examinations were performed by two independent investigators that were blinded to dog identification and clinical and parasitological data.

### Statistical analysis

Results were analysed using univariate statistical analyses (i.e. non-parametric Wilcoxon signed- rank test and t-test) to determine the level of significant differences between pre- (Day − 15) and post-treatment (at Day 180 and at Day 270) based on the antigen status, micofilarial counts and radiological and cardiac scores in the three treatment groups (G1, G2 and G3). Statistical analysis was performed using Windows SPSS® (version 17.0). The level of significance was set at a *P* value < 0.05.

## Results

### Parasitological findings

Table 1 summarizes the results of Knott’s test of all dogs at enrolment, while Table 2 reports results of antigen testing scores. All dogs except three were positive for circulating mff, with values ranging from 50 to 83,600 mff/ml blood. Circulating antigens were present in all but two dogs, who were however positive for circulating microfilariae. Antigen levels were comparable among dogs of the three groups.

**Table 1:** *Dirofilaria immitis* microfilarial count at enrolment (mff/ml) evaluated through Knott’s test

Dog number	Group 1 Moxidectin oral	Dog number	Group 2 Moxidectin Spot-On	Dog number	Group 3 Moxidectin injectable
1	0	1	62,500	1	39,400
2	2500	2	0	2	13,800
3	2900	3	1100	3	100
4	10,150	4	22,800	4	15,900
5	200	5	3100	5	8600
6	200	6	40,800	6	3900
7	0	7	5600	7	200
8	50	8	70,350	8	11,200
9	0	9	83,600	9	31,500
10	200	10	33,800	10	12,200
Average value ± SD	1620 ± 3190.6		31,810.6 ± 31,733.1		13,680 ± 12,823.3
Minimum and maximum value	0–10,150		0–83,600		200–39,400
95%CI	130–3664		13,815–51,304		6710–21,739

**Table 2:** Circulating antigen scores at enrolment (Days –15)

Dog number	Group 1 Moxidectin oral	Dog number	Group 2 Moxidectin Spot-On	Dog number	Group 3 Moxidectin injectable
1	+ +	1	+ +	1	+ +
2	+ +	2	+ +	2	+ +
3	+ +	3	+	3	-
4	+ +	4	+ +	4	+ +
5	+ +	5	+ +	5	-
6	+ +	6	+ +	6	+ +
7	+ +	7	+ +	7	+ +
8	+ +	8	+ +	8	+ +
9	+ +	9	+	9	+ +
10	+ +	10	+ +	10	+ +

Negative (−), weak positive (+) and strong positive (+ +)

Twenty-two/30 dogs (73.3%) were also positive for *D. repens* mff (range 100–78,200 mff/ml).

**Fig. 2:**
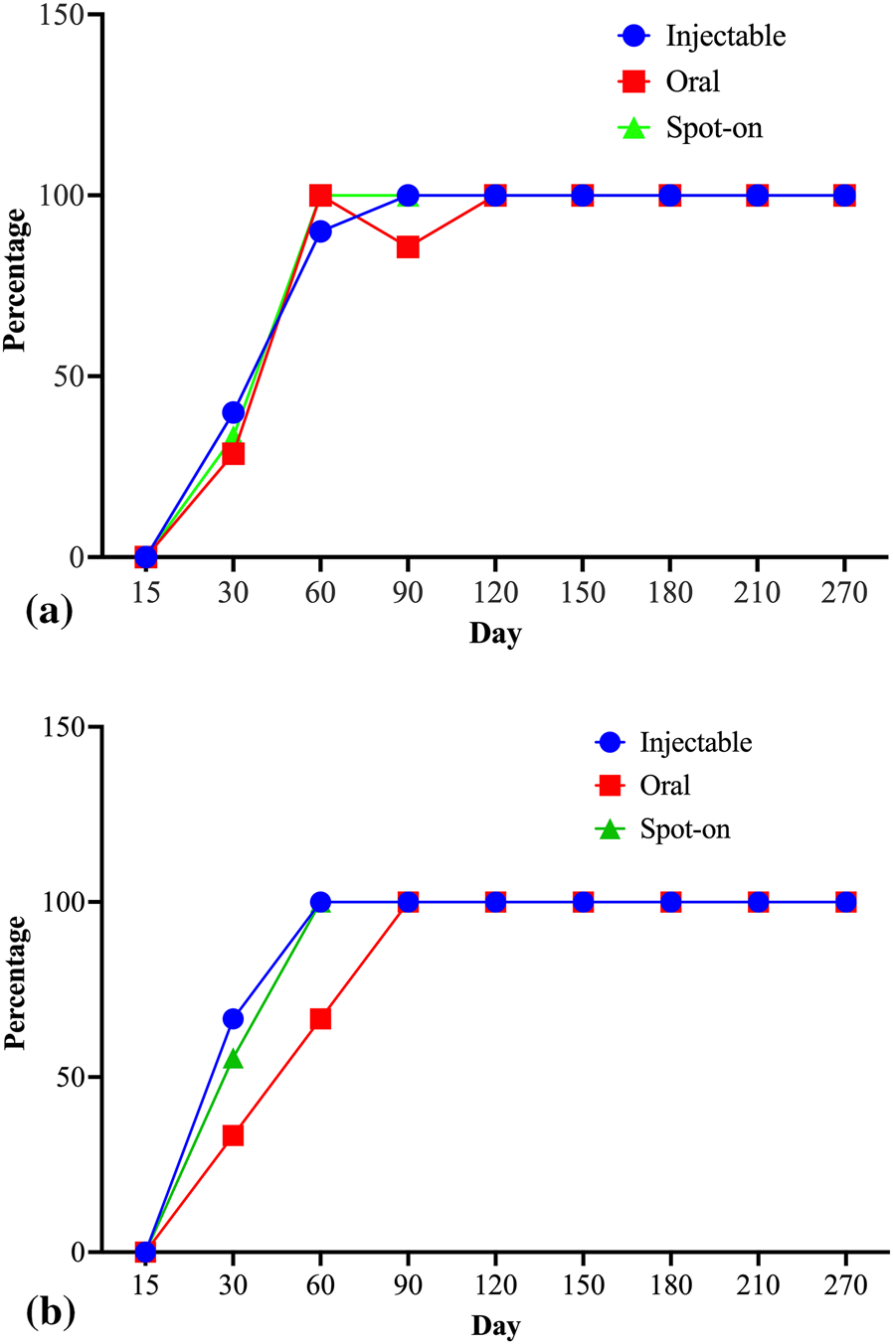
Reduction of circulating microfilariae of *D. immitis* (**a**) and *D. repens* (**b**) in the three treatment groups

Figure 2a and Table 3 report the results for reduction of circulating *D. immitis* microfilariae induced by the three treatment protocols evaluated in the study. In general, mff loads decreased markedly in all dogs from all groups at Day 30, and all but one dog were negative at Day 60. The one positive dog remaining was from the injectable formulation group (G3) and became negative at Day 90. Interestingly, dog no. 3 from the oral formulation group (G1) became negative at Day 60 (from 2900 mff/ml at enrolment) and then became once again positive for mff at Day 90 (100 mff/ml). The dog was negative at all subsequent time points.

**Table 3:** Number of microfilarie counted at different time points (from Day − 15 to Day 270) evaluated through Knott’s test

Dog number	Moxy		mff/ml Day− 15	mff/ml Day 30	mff/ml Day 60	mff/ml Day 90	mff/ml Day 120 to Day 270
1	Injectable	*D. immitis*	39,400	50	0	0	0
		*D. repens*	8600	0	0	0	0
2		*D. immitis*	13,800	700	0	0	0
		*D. repens*	7600	350	0	0	0
3		*D. immitis*	100	0	0	0	0
		*D. repens*	15,200	0	0	0	0
4		*D. immitis*	15,900	3600	100	0	0
		*D. repens*	0	0	0	0	0
5		*D. immitis*	8600	50	0	0	0
		*D. repens*	10,300	0	0	0	0
6		*D. immitis*	3900	0	0	0	0
		*D. repens*	2000	0	0	0	0
7		*D. immitis*	200	0	0	0	0
		*D. repens*	1550	0	0	0	0
8		*D. immitis*	11,200	0	0	0	0
		*D. repens*	13,600	0	0	0	0
9		*D. immitis*	31,500	600	0	0	0
		*D. repens*	16,000	100	0	0	0
10		*D. immitis*	12,200	250	0	0	0
		*D. repens*	55,000	100	0	0	0
1	Oral	*D. immitis*	0	0	0	0	0
		*D. repens*	0	0	0	0	0
2		*D. immitis*	2500	400	0	0	0
		*D. repens*	0	100	0	0	0
3		*D. immitis*	2900	1550	0	100	0
		*D. repens*	0	0	0	0	0
4		*D. immitis*	10,150	2750	0	0	0
		*D. repens*	0	0	0	0	0
5		*D. immitis*	200	50	0	0	0
		*D. repens*	11,750	4900	200	0	0
6		*D. immitis*	200	0	0	0	0
		*D. repens*	0	0	0	0	0
7		*D. immitis*	0	0	0	0	0
		*D. repens*	100	0	0	0	0
8		*D. immitis*	50	0	0	0	0
		*D. repens*	150	0	0	0	0
9		*D. immitis*	0	0	0	0	0
		*D. repens*	0	0	0	0	0
10		*D. immitis*	200	50	0	0	0
		*D. repens*	0	0	0	0	0
1	Spot-On	*D. immitis*	62,500	350	0	0	0
		*D. repens*	28,400	0	0	0	0
2		*D. immitis*	0	0	0	0	0
		*D. repens*	0	0	0	0	0
3		*D. immitis*	1100	0	0	0	0
		*D. repens*	21,200	0	0	0	0
4		*D. immitis*	22,800	250	0	0	0
		*D. repens*	3350	50	0	0	0
4		*D. immitis*	3100	0	0	0	0
		*D. repens*	8700	0	0	0	0
6		*D. immitis*	40,800	200	0	0	0
		*D. repens*	14,200	50	0	0	0
7		*D. immitis*	5600	100	0	0	0
		*D. repens*	3100	50	0	0	0
8		*D. immitis*	70,350	200	0	0	0
		*D. repens*	9350	50	0	0	0
9		*D. immitis*	83,600	0	0	0	0
		*D. repens*	78,200	0	0	0	0
10		*D. immitis*	33,800	150	0	0	0
		*D. repens*	8200	0	0	0	0

All dogs with *D. repens* mff were negative at Day 90 (Fig. 2b).

Table 4 reports the results for antigen testing for each group at each time point until Day 270. There was a clear reduction of antigen concentration for all treatment groups, beginning as early as Day 60. Overall, negative antigen results (at least two consecutive negative tests) were observed at Day 60 for 2 dogs; at Day 150 for 1 dog; at Day 180 for 4 dogs; at Day 210 for 10 dogs; at Day 240 for 4 dogs. At Day 270, 9/10 dogs (90.0%) from G1, 6/10 dogs (60.0%) from G2 and 8/10 dogs (80.0%) from G3 had at least two consecutive negative tests. Several dogs from each group reverted to positive antigen status at different time points following negativization.

**Table 4:**
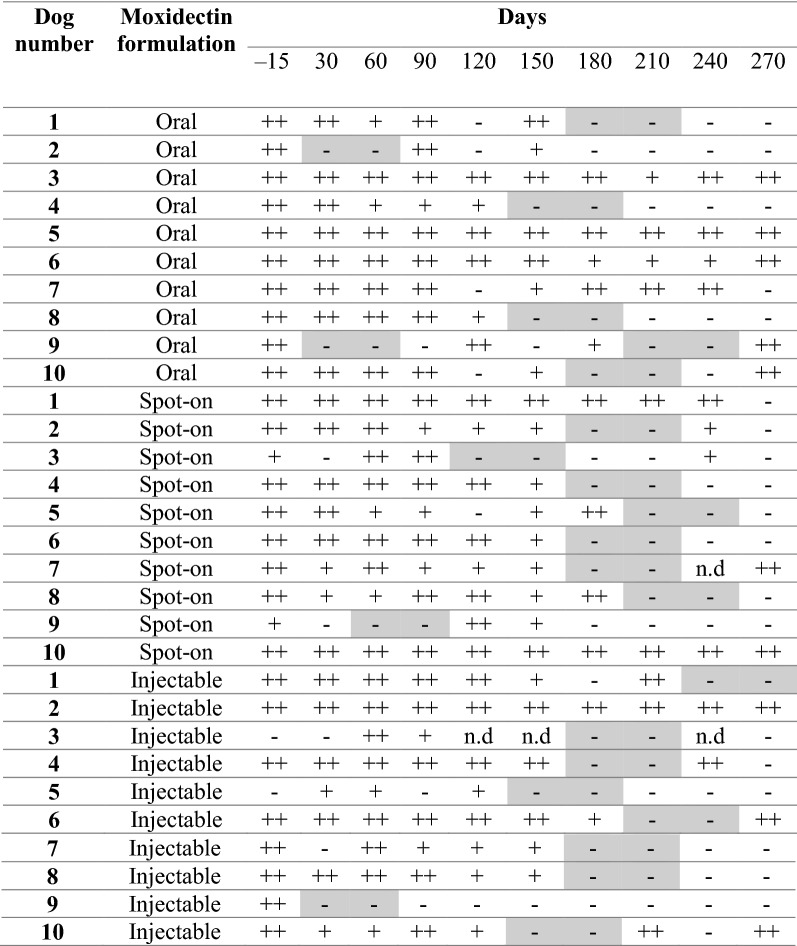
Results of the antigen testing for each treatment groups (G1, G2 and G3) from Day 30 to Day 270

The results of statistical analyses showed a significant difference in antigen status before and after treatment in all three groups (G1: *P* = 0.015; G2: *P* = 0.006; G3: *P* = 0.020). However, the paired sample t-test revealed no significant difference among the three groups after treatment on Day 270 (*P* > 0.05). In addition, there was a significant difference between pre- and post-treatment in terms of mff counts in all three groups starting at Day 30 through Day 270 (*P* < 0.05).

### Radiological and cardiac ultrasound findings

Table 5 reports the radiological and cardiopulmonary scores obtained by all the dogs in the three treatment groups before treatment (Day − 15) and after treatment (Day 180). A more detailed description of pulmonary and/or cardiac alterations can be found as Additional file 1: Table S1.

**Table 5:**
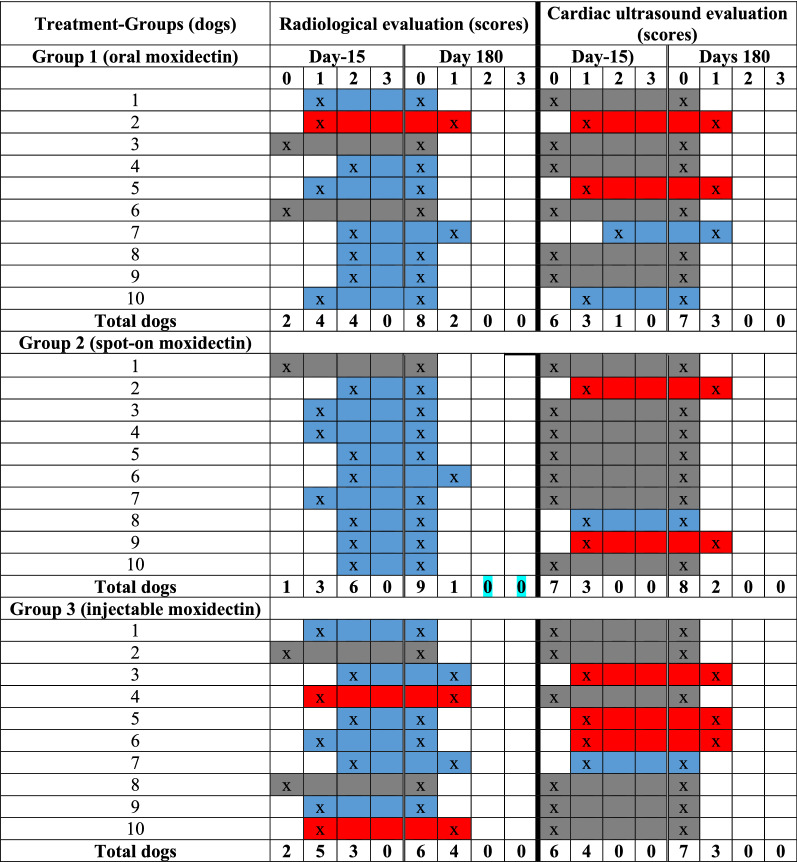
Results of radiological and cardiac ultrasound evaluation (scores) for all the dogs in each treatment groups (G1, G2, G3) at Day − 15 (pre-treatment) and at Day 180 (post-treatment)

Colour: Blue: Improvement (score 2 to score 1; score 1 to score 0; score 2 to score 0), Red: Without changes (score 1 to score 1; score 2 to score 2), Grey: Normal (score 0 to score 0)

The results of radiological examination showed that at Day − 15, 5/30 (16.6%) were assigned a score of 0 (2 dogs from G1; 1 dog from G2; 2 dogs from G3), 12/30 (40%) a score of 1 (four dogs from G1; three dogs from G2; five dogs from G3) and 13/30 (43.4%) a score of 2 (four dogs from G1; six dogs from G2; three dogs from G3). No dog was assigned to score 3.

**Fig. 3:**
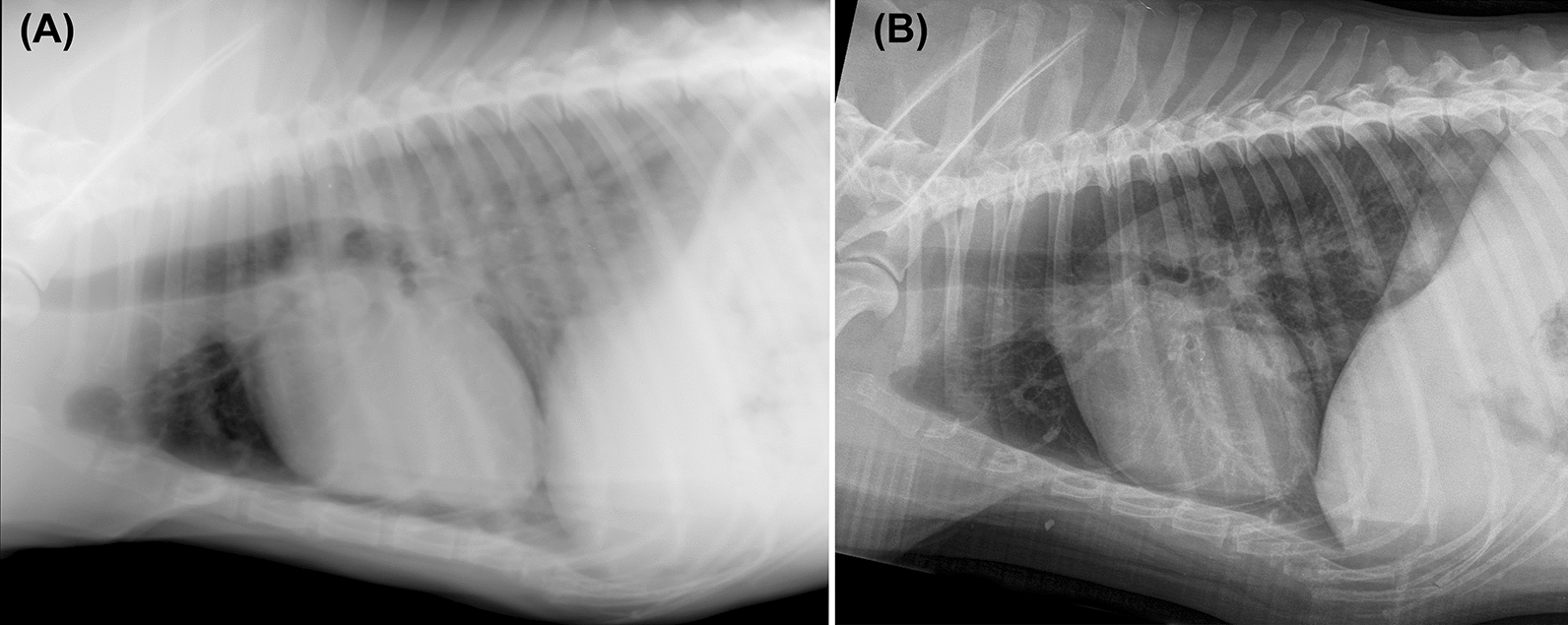
Right-lateral radiographs pre- (**A**) and post-treatment (**B**) of the same dog. **A** A diffuse mixed alveolar/unstructured interstitial pattern. This increase in pulmonary opacity involves especially the caudal lung lobes where multiple air bronchograms are visible. In **B**, there is a partial resolution of the radiographic alterations involving the lung lobes, although there is a residual unstructured interstitial pattern associated with mild to moderate thickening of the bronchial walls. This dog was initially assigned in score 2, but after the 6 months of therapy was assigned a score of 1

**Fig. 4:**
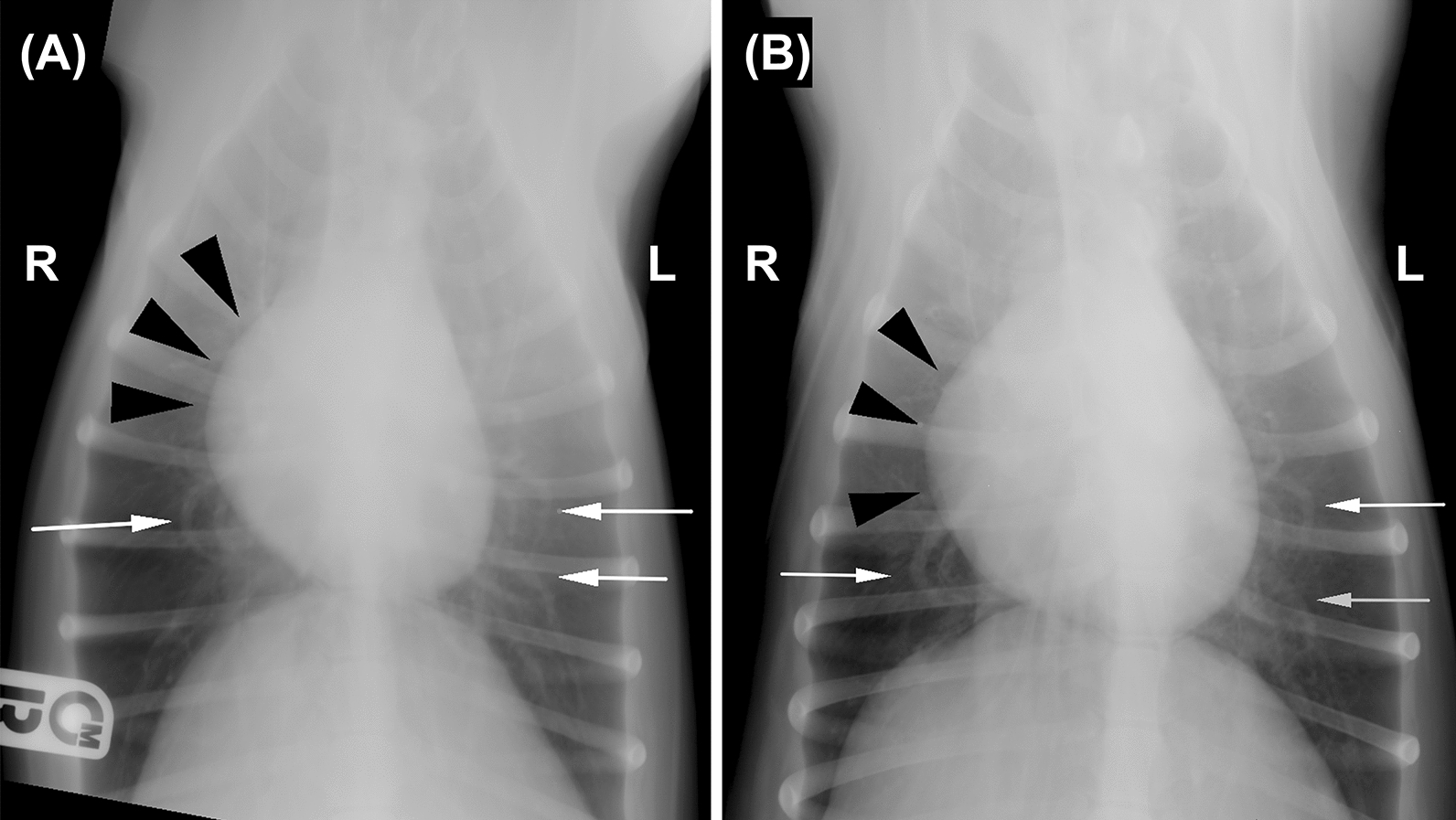
Dorsoventral (DV) radiographs pre- (**A**) and post-treatment (**B**) of the same dog. In both views, there is a mild, diffuse, unstructured interstitial pattern associated with mild dilatation of the right atrium (black arrowheads). Furthermore, several caudal lobar arteries (white arrows) have a convoluted shape. This dog was assigned a pre-treatment score of 1 and, after 6 months of therapy, was re-assigned in the same score. Legend: R = right, L = left

At Day 180, all the dogs with score 0 at day − 15 were assigned the same score. Furthermore, 9/12 (75%) dogs with score 1 at Day − 15 showed substantial improvement at Day 180 (Fig. 3) and the remaining three were stable (one dog from G1 and two dogs from G3; Fig. 4). The 13 dogs with score 2 at day − 15 showed radiographic improvement of pulmonary and/or cardiac conditions. Indeed, 4/13 (30.7%) dogs had a partial resolution of the radiological alterations and were reduced to score 1 (one dog from G1; one dog from G2 and two dogs from G3) and 9/13 (69.3%) had a substantial improvement and therefore were moved from score 2 into score 0 (three dogs from G1; five dogs from G2 and one dog from G3).

Cardiac ultrasound examination showed that at the beginning of the study (Day − 15), 11/30 (36.7%) dogs showed altered pulmonary blood flow, ranging from score 1 (three dogs from G1; three dogs from G2; four dogs from G3) to score 2 (one dog from G3). Moreover, 19/30 (63.3%) dogs were assigned score 0 (six dogs from G1; seven dogs from G2 and six dogs from G3). In particular, 12/30 (40%) dogs were classified with chronic degenerative mitral disease (CDMD), representing a comorbidity for the enrolled animals [26]. Specifically, two of the dogs classified with score 1, one dog with score 2 and three dogs with score 0 showed enlarged left atrium and concomitant haemodynamic significant mitral regurgitation and were classified in stage B2 of CDMD. Moreover, stage B1 of CDMD was recorded in five dogs classified in score 0 and in three dogs classified in score 1. Six dogs with CDMD in stage B2 (6/30) received oral administration of benazepril 0.25 mg/kg SID.

Subsequent echocardiographs carried out at day 180 did not show any significant variation of previously assigned scores, except for 4/11 dogs that showed a progressive improvement. In particular, three dogs with slight alteration assigned in score 1 became normal (one dog from each group) while one dog (from G1) with moderate alteration assigned a score of 2 showed slight modifications and was assigned a score of 1 (Fig. 4). However, according to the American College of Veterinary Internal Medicine (ACVIM) guidelines [27], the echocardiographic probability of pulmonary hypertension (PH) was low (only one dog staged in score 2) considering that peak tricuspid regurgitation velocity was ≤ 3.0. During the second echocardiographic control, PH score and peak tricuspid regurgitation velocity were reduced.

Results of statistical analysis of radiological scores obtained before treatment (Day − 15) and after treatment (Day 180) showed that treatment with moxidectin/doxycycline was effective for all the animals in the three treatment groups (*P* = 0.000). There was no significant difference among the three groups (*P* = 0.564) post-treatment at Day 180. Regarding the cardiac evaluation, the Wilcoxon test showed statistically significantly higher rank of post-treatment scores than the pre-treatment rank scores (*P* = 0.045).

## Discussion

In the present study we evaluated the efficacy of different formulations of moxidectin combined with doxycycline to induce negative antigen status in dogs naturally infected with *D. immitis*. According to [28], even though melarsomine is still widely used by veterinary practitioners in Italy, a monthly macrocyclic lactone together with doxycycline is currently being used by over 30% of surveyed veterinary facilities. The use of moxidectin is a valid alternative to ivermectin, as shown by several studies [8–10, 17, 29].

Microfilarial counts were not considered when forming the different treatment groups. It is known that the intensity of microfilaraemia is not correlated with the adult worm burden [30].

The adulticidal efficacy (i.e. two consecutive negative antigen tests) of the injectable formulation of moxidectin was 90% at 9 months, slightly higher compared to the Spot-On formulation (80%) and the oral formulation (60%). This may be due to its pharmacokinetics. Lok et al. [31], in an early pharmacokinetic study of the formulation used here (0.17 mg moxidectin/kg), reported that effective serum moxidectin levels peak 8 days after injection and remain at this level for 6 months. McCall et al. [32] studied the retroactive activity of moxidectin extended release on immature worms and reported 85.9% efficacy against 4-month-old *D. immitis* infections and efficacy was even higher (97.2%) when a second treatment was given 6 months later. Only one previous study assessed the efficacy of an extended-release injectable formulation of moxidectin combined with doxycycline, in which two doses 6 months apart resulted in 90% of dogs becoming antigen negative [18].

The Spot-On formulation was efficacious in 80% of dogs at 9 months. Bowman et al. [33] reported that topical moxidectin reaches steady-state serum concentrations at levels that are much higher than those needed for heartworm prophylaxis. The exposure of adult parasites to high concentrations of moxidectin likely contributes to the efficacy of this protocol.

Oral moxidectin combined with doxycycline gave the lowest percentage of dogs (60%) that became negative for circulating antigens by 9 months. It has been reported that, when compared to oral ivermectin, moxidectin has lower total body clearance and higher volume of distribution, which results in a prolonged elimination half-life [34]. However, it may be that oral administration is not always followed by optimal gastrointestinal absorption, leading to under-dosing and lack of efficacy. It is well known that bioavailability of orally administered drugs depends on a multitude of factors, including gastric pH and emptying time, small intestinal fluid properties, changes in gastrointestinal integrity, etc. [35].

The concentration of circulating antigens was variable from month to month in all treatment groups, as reported previously by others [8–10], and the authors of the present study defined “first negative” as a minimum of two consecutive negative tests, according to [10]. The reason for transitory return to antigen-positive status is not clear, but may be due to various factors including variable antigen concentrations due to gradual death of parasites or the arrival of migrating worms to the pulmonary artery during the study period. This phenomenon, however, may not be unique to alternative protocols using doxycycline and macrocyclic lactones. Paterson et al. [10], who compared doxycycline/moxidectin to melarsomine, reported variations in the results of monthly antigen testing in both groups. In a similar study [9] authors reported a return to positive antigen status in one dog treated with melarsomine at 12 months post-treatment. To the authors’ knowledge, no other studies have evaluated monthly antigen status in dogs treated with melarsomine. Early studies only evaluated dogs after 4, 8 or 12 months following treatment and also reported persistent antigenemia in some dogs [36–38]. It would be interesting to conduct studies on the monthly trend of antigen status following the diverse adulticide regimens.

It has also been reported that immune-complex formation can lead to false-negative antigen test results and that this happens frequently in dogs treated with doxycycline and macrocyclic lactones. Pre-heating serum samples can disrupt immune-complexes, resulting in a positive test result [39, 40]. The authors of the present study chose not to preheat the serum samples. Over 70% of dogs were also infected with *D. repens*, and it has been reported that pre-heating serum samples from *D. repens* mono-infected dogs leads to false-positive antigen tests for *D. immitis* [41, 42]. Correct interpretation of conversion to positive antigen status in these dogs would have been impossible. Furthermore, according to the American Heartworm Society guidelines for canine heartworm disease, heat treatment should be considered only when antigen test is negative, but mff are circulating [15]: in the present study only two dogs were mff positive and antigen negative and both were positive for *D. repens*.

Treatment regimens were also effective against *D. repens* mff, with no differences observed among the three formulations used (data not shown). However, there is no current test to verify adulticide efficacy against *D. repens*, and the clearance of circulating mff observed in the present study is not necessarily indicative of adult worm death. However, Petry et al. [43] reported the adulticide effect of the same topical formulation used in the present study in *D. repens* experimentally infected dogs.

It has been reported that dogs treated with doxycycline can have gastrointestinal upset, while coughing has also been reported in dogs treated with the ML/doxycyline protocol [10]. In the present study, all treatment regimens were well tolerated. Only six dogs were treated with furosemide and benazepril for cough and mitral regurgitation during the entire study.

Radiographic alterations occurring during natural heartworm infection are related to the parasite load and time elapsed since the infection, with radiographic findings ranging from subclinical disease without apparent alterations in the lung fields and pulmonary vasculature to severe pulmonary and cardio-circulatory impairment [44–46]. In our study, the most frequent radiographic findings in dogs with score 1 were a diffuse interstitial pattern and pulmonary vascular changes, similar to those reported by Mavrapoulou et al. and Genchi et al. [6, 9]. These findings are considered common in cases of heartworm disease, and the alterations in the lung parenchyma are attributed to eosinophilic bronchopneumonia, fibrotic changes and focal pulmonary consolidation [44, 47].

The dogs classified as moderate (score 2) had dilatation of the right atrium, among the most frequent alterations, likely related to infection with a higher or long-lasting parasite load and a more severe pulmonary interstitial pattern. Moreover, there were many dogs with enlargement of the left atrium or an overall increase in cardiac size, most likely related to concurrent mitral valve disease.

In our study, there was no evidence of worsening of the radiographic findings at the follow-up after 6 months. Moreover, four of the dogs initially classified in score 2 showed a partial improvement and nine an almost complete resolution. Similarly, all the dogs with score 1 improved. Overall, the treatment with moxidectin and doxycycline combination was effective and almost all the dogs from the treatment groups were cleared of pulmonary abnormalities by 6 months from the beginning of treatment. The combination of moxy/doxy was previously reported in experimentally treated animals to induce the reduction of pro-inflammatory antigen mass [14]. Similarly, Genchi et al. [9] reported that no dogs showed worsening of pulmonary patterns 12 to 24 months after the treatment with the same topical formulation of moxidectin combined with doxycycline for the first 30 days in dogs naturally infected by *D. immitis*.

Although echocardiography represents a valid option for measurement of pulmonary artery pressure (PAP), limits of the method include variability and imprecision in individual dogs. Therefore, evaluation of echocardiographic Doppler parameters is useful for assessing the probability, rather than the diagnosis, of pulmonary hypertension (PH), as shown by Reinero et al. [27]. However, in our study, echocardiography gave important information regarding the diagnostic profile and the therapeutic follow-up. Echocardiography showed a progressive improvement of cardiac function in a limited number of animals (4/30). Notably, we observed CDMD as a comorbidity associated with heartworm disease in 40% of dogs enrolled and this could have influenced assignment of echocardiographic scores [48].

## Conclusions

In conclusion, doxycycline/moxidectin combination treatment for HWD has been reported as being safe and effective. Results from the present study suggest that efficacy may be related to the moxidectin formulation.

## Supplementary Information

**Additional file 1: Table S1.** Results of pulmonary and/or cardiac alterations (left atrial enlargement; pulmonary arteries enlargement; interstitial pattern; cardiac enlargement; right atrial enlargement; bronchial pattern; main pulmonary artery enlargement; alveolar pattern) for both radiographic evaluations (day – 15 and day 180) for each dog from all the treatment-groups (oral, Spot-On, injectable).

## Data Availability

All data obtained are shown in the manuscript.
