# Supplementary material for: Efficacy of oral, topical and extended-release injectable formulations of moxidectin combined with doxycycline in Dirofilaria immitis naturally infected dogs
Source: Parasit Vectors. 2023 Feb 6;16:54. doi: 10.1186/s13071-023-05673-9 (PMC9901089; doi:10.1186/s13071-023-05673-9)
Supplement: Supplementary file 1 — Additional file 1: Table S1. Results of pulmonary and/or cardiac alterations (left atrial enlargement; pulmonary arteries enlargement; interstitial pattern; cardiac enlargement; right atrial enlargement; bronchial pattern; main pulmonary artery enlargement; alveolar pattern) for both radiographic evaluations (day – 15 and day 180) for each dog from all the treatment-groups (oral, Spot-On, injectable). [file 13071_2023_5673_MOESM1_ESM.docx]

Table S1. Results of pulmonary and/or cardiac alterations (left atrial enlargement; pulmonary arteries enlargement; interstitial pattern; cardiac enlargement; right atrial enlargement; bronchial pattern; main pulmonary artery enlargement; alveolar pattern) for both radiographic evaluations (day -15 and day 180) for each dog from all the treatment-groups (oral, spot-on, injectable).

| DAY -15 | | | | | | | | | | | | | | | | DAY 180 | | | | | | |
| --- | --- | --- | --- | --- | --- | --- | --- | --- | --- | --- | --- | --- | --- | --- | --- | --- | --- | --- | --- | --- | --- | --- |
| **Dog** | SCORE 0 | SCORE 1 | | | | | | SCORE 2 | | | | | | | | SCORE 0 | SCORE 1 | | | | | |
|  | **N** | **LAE (Mi)** | **IP (Mi)** | **PAE (Mi)** | **CE (Mi)** | **RAE (Mi)** | **BP (Mi)** | **LAE (Mo)** | **IP (Mo)** | **PAE (Mo)** | **CE (Mo)** | **RAE (Mo)** | **MPAE** | **BP (Mi)** | **AP** | **N** | **LAE (Mi)** | **IP (Mi)** | **PAE (Mi)** | **CE (Mi)** | **RAE (Mi)** | **BP (Mi)** |
| **Group 1 ORAL** |  |  |  |  |  |  |  |  |  |  |  |  |  |  |  |  |  |  |  |  |  |  |
| 1 |  | x |  | x |  |  |  |  |  |  |  |  |  |  |  | x |  |  |  |  |  |  |
| 2 |  | x |  | x |  |  |  |  |  |  |  |  |  |  |  |  | x |  | x |  |  |  |
| 3 | x |  |  |  |  |  |  | x |  | x |  |  |  |  |  | x |  |  |  |  |  |  |
| 4 |  |  |  |  |  |  |  | x |  | x |  |  |  |  |  | x |  |  |  |  |  |  |
| 5 |  | x | x | x |  |  |  |  |  |  |  |  |  |  |  | x |  |  |  |  |  |  |
| 6 | x |  |  |  |  |  |  |  |  |  |  |  |  |  |  | x |  |  |  |  |  |  |
| 7 |  | x |  |  |  |  |  |  | x | x |  |  |  |  |  |  |  | x | x |  | x |  |
| 8 |  |  |  |  |  | x |  | x | x | x |  |  |  |  |  | x |  |  |  |  |  |  |
| 9 |  | x |  |  |  |  |  |  | x | x |  |  | x |  |  | x |  |  |  |  |  |  |
| 10 |  | x | x |  |  |  |  |  |  |  |  |  |  |  |  | x |  |  |  |  |  |  |
| **Group 2**  **SPOT-ON** |  |  |  |  |  |  |  |  |  |  |  |  |  |  |  |  |  |  |  |  |  |  |
| 1 | x |  |  |  |  |  |  |  |  |  |  |  |  |  |  | x |  |  |  |  |  |  |
| 2 |  |  |  |  |  |  |  |  | x | x |  | x |  |  | x |  |  | x | x |  |  | x |
| 3 |  | x | x | x |  |  |  |  |  |  |  |  |  |  |  | x |  |  |  |  |  |  |
| 4 |  | x | x | x |  |  |  |  |  |  |  |  |  |  |  | x |  |  |  |  |  |  |
| 5 |  | x |  |  |  |  |  |  | x | x |  | x |  |  |  | x |  |  |  |  |  |  |
| 6 |  | x |  |  |  |  |  |  | x | x |  |  |  | x |  |  | x | x | x | x |  |  |
| 7 |  | x | x | x | x |  |  |  |  |  |  |  |  |  |  | x |  |  |  |  |  |  |
| 8 |  |  |  |  |  |  |  | x | x | x |  |  |  |  | x | x |  |  |  |  |  |  |
| 9 |  |  |  |  |  |  |  |  |  | x | x |  |  |  |  | x |  |  |  |  |  |  |
| 10 |  |  |  |  |  |  |  |  |  | x |  |  | x |  |  | x |  |  |  |  |  |  |
| **Group 3**  **INJECTABLE** |  |  |  |  |  |  |  |  |  |  |  |  |  |  |  |  |  |  |  |  |  |  |
| 1 |  | x | x |  |  |  |  |  |  |  |  |  |  |  |  | x |  |  |  |  |  |  |
| 2 | x |  |  |  |  |  |  |  |  |  |  |  |  |  |  | x |  |  |  |  |  |  |
| 3 |  | x |  |  | x |  |  |  | x | x |  |  |  |  |  |  | x | x |  |  |  | x |
| 4 |  | x |  | x |  |  | x |  |  |  |  |  |  |  |  |  | x |  |  | x |  | x |
| 5 |  |  |  |  |  |  |  |  |  | x |  |  | x | x |  | x |  |  |  |  |  |  |
| 6 |  | x | x |  |  |  |  |  |  |  |  |  |  |  |  | x |  |  |  |  |  |  |
| 7 |  |  |  |  |  |  |  | x |  | x |  |  |  |  |  |  |  |  |  |  |  |  |
| 8 | x |  |  |  |  |  |  |  |  |  |  |  |  |  |  | x |  |  |  |  |  |  |
| 9 |  | x | x | x | x |  |  |  |  |  |  |  |  |  |  | x |  |  |  |  |  |  |
| 10 |  |  | x |  |  | x |  |  |  |  |  |  |  |  |  |  |  | x |  |  |  |  |

**N**: normal; **Mi**: mild; **Mo**: moderate; **LAE**: left atrial enlargement; **PAE**: pulmonary arteries enlargement; **IP**: interstitial pattern; **CE**: cardiac enlargement; **RAE**: right atrial enlargement; **BP**: bronchial pattern; **MPAE**: main pulmonary artery enlargement; **AP**: alveolar pattern
